# A global perspective of advanced practice nursing research: A review of systematic reviews protocol

**DOI:** 10.1371/journal.pone.0280726

**Published:** 2023-01-24

**Authors:** Kelley Kilpatrick, Isabelle Savard, Li-Anne Audet, Abby Kra-Friedman, Renée Atallah, Mira Jabbour, Wentao Zhou, Kathy Wheeler, Elissa Ladd, Deborah C. Gray, Colette Henderson, Lori A. Spies, Heather McGrath, Melanie Rogers

**Affiliations:** 1 Susan E. French Chair in Nursing Research and Innovative Practice, Ingram School of Nursing, Faculty of Medicine and Health Sciences, McGill University, Montréal, Québec, Canada; 2 Centre intégré universitaire de santé et de services sociaux de l’Est-de-l’Île-de-Montréal (CIUSSS-EMTL), Montréal, Québec, Canada; 3 Ingram School of Nursing, Faculty of Medicine and Health Sciences, McGill University, Montréal, Québec, Canada; 4 Henrietta Szold School of Nursing, Faculty of Medicine, Hebrew University of Jerusalem, Hadassah Ein Kerem, Jerusalem, Israel; 5 School of Nursing, Duquesne University, Pittsburgh, Pennsylvania, United States of America; 6 Alice Lee Centre for Nursing Studies, Yong Loo Lin School of Medicine, National University of Singapore, National University Health System, Singapore, Singapore; 7 College of Nursing, University of Kentucky, Lexington, Kentucky, United States of America; 8 School of Nursing, MGH Institute of Health Professions, Boston, Massachusetts, United States of America; 9 School of Nursing, Old Dominion University, Virginia Beach, Virginia, United States of America; 10 School of Health Sciences, University of Dundee, Dundee, Scotland, United Kingdom; 11 Louise Herrington School of Nursing, Baylor University, Dallas, Texas, United States of America; 12 St James Public Health Services, Montego Bay, St James, Jamaica; 13 Department of Nursing and Midwifery, University of Huddersfield, Queensgate, Huddersfield, United Kingdom; Charles Darwin University, AUSTRALIA

## Abstract

**Introduction:**

In 2020, the World Health Organization called for the expansion and greater recognition of all nursing roles, including advanced practice nurses (APNs), to better meet patient care needs. As defined by the International Council of Nurses (ICN), the two most common APN roles include nurse practitioners (NPs) and clinical nurse specialists (CNSs). They help ensure care to communities as well as patients and families with acute, chronic or complex conditions. Moreover, APNs support providers to deliver high quality care and improve access to services. Currently, there is much variability in the use of advanced practice nursing roles globally. A clearer understanding of the roles that are in place across the globe, and how they are being used will support greater role harmonization, and inform global priorities for advanced practice nursing education, research, and policy reform.

**Objective:**

To identify current gaps in advanced practice nursing research globally.

**Materials and methods:**

This review of systematic reviews will provide a description of the current state of the research, including gaps, on advanced practice nursing globally. We will include reviews that examine APNs, NPs or CNSs using recognized role definitions. We will search the CINAHL, EMBASE, Global Health, HealthStar, PubMed, Medline, Cochrane Library Database of Systematic Reviews and Controlled Trials Register, Database of Abstracts of Reviews of Effects, Joanna Briggs Institute, and Web of Science electronic databases for reviews published from January 2011 onwards, with no restrictions on jurisdiction or language. We will search the grey literature and hand search the reference lists of all relevant reviews to identify additional studies. We will extract country, patient, provider, health system, educational, and policy/scope of practice data. We will assess the quality of each included review using the CASP criteria, and summarize their findings. This review of systematic reviews protocol was developed following the PRISMA-P recommendations.

**PROSPERO registration number:**

CRD42021278532.

## Introduction

In 2020, the World Health Organization [1] called for the expansion and greater recognition of all nursing roles, including nurses in advanced practice, to better meet patient care needs. Nurses in advanced practice roles, as defined by the International Council of Nurses (ICN), are most often identified as advanced practice nurses (APNs), with the two most common APN roles being nurse practitioners (NPs) and clinical nurse specialists (CNSs) [2]. They help ensure care to communities as well as patients and families with acute, chronic or complex conditions [2]. In addition to providing direct care, NPs and CNSs support care providers to deliver high quality care and improve access to services [3–5]. Nurses in these roles have educational preparation at the Master’s level or above in addition to in-depth clinical expertise and complex decision-making skills [6]. A global analysis of advanced practice nursing policy, regulation and practice by Ladd et al. [7] highlighted that advanced practice nursing roles are growing at an accelerated rate. However, these authors argue that advanced practice nursing roles have emerged unequally across the globe in response to local care needs without clear supports to develop consistent expanded roles for nurses. A recent review of systematic reviews of primary healthcare NP roles identified 396 primary studies included in the 40 systematic reviews representing on average 3 countries (range not reported to 9) [8]. Although there are several systematic reviews of APN and CNS roles in other clinical settings [4, 5, 9], no synthesis of this body of evidence is available for other recognized advanced practice roles, making it challenging to compare advanced practice nursing roles across jurisdictions.

Currently, there is much variability in the use of advanced practice nursing roles globally [1, 7, 10, 11]. A clearer understanding of the roles that are in place across the globe, how they are being used and the outcomes that are being assessed would support greater role harmonization, and inform global priorities for advanced practice nursing education, research, and policy reform.

To identify current gaps in advanced practice nursing research globally, we propose to conduct of review of systematic reviews of studies examining APNs, NPs or CNSs using recognized advanced practice nursing role definitions [2]. We will seek to answer the question: Do current systematic reviews that include APNs, NPs or CNSs represent countries where these roles are found globally? To do so, we will address the following three aims:

1. Identify the countries included in systematic reviews of APNs, NPs or CNSs;
2. Describe the types of included studies, study population, role definitions, and context of care identified in the systematic reviews; and
3. Examine the types of outcomes of APN, NP or CNS roles included in systematic reviews globally.

## Materials and methods

This review of systematic reviews will provide a description of the current state of the research, including gaps, on advanced practice nursing globally. We adapted methods used in an umbrella review that sought to identify indicators sensitive to the practice of primary healthcare NP practice [12]. The protocol for the review of reviews was developed following the PRISMA-P recommendations by Shamseer et al. [13]. The review of reviews is registered with the PROSPERO International Prospective Register of Systematic Reviews (Prospero ID CRD42021278532).

### Inclusion criteria

#### Types of studies

We will include all relevant published and unpublished systematic reviews reported from January 2011 onwards, with no restrictions on jurisdiction or language. For a review to be identified as systematic, a specific research question must be present or sufficient information must be provided so reviewers can identify the components of a research question (i.e., PICOS) related to advanced practice nursing. Additionally, the review must use prespecified inclusion and exclusion criteria, as well as systematic methods to identify relevant published and unpublished evidence to minimize the risk of bias in the retained studies [14]. Systematic reviews will be included provided the advanced practice nursing role is clearly defined and the APN, NP or CNS has decision-making autonomy [2].

#### Types of participants

Participants will include patients and providers. Patients of any age, health condition, groups or communities receiving care from an APN, NP or CNS in all types (e.g., public/private; teaching/non-teaching,), sizes (e.g., small/medium/large) and locations (e.g., urban/rural) of community or care agencies (e.g., acute, long-term care, primary care, home care) will be retained. Providers will include all members of the healthcare team in all types, sizes, and locations of organizations. We will extract data to describe the country, number of participants, patient health conditions (e.g., diabetes, mental health), type of care (e.g., post-operative care), organizational characteristics, provider roles in the team, reason of APN, NP or CNS intervention (e.g., educational offering), and type of outcome.

#### Types of interventions

We will include studies of APNs, NPs or CNSs in all sectors. To capture the countries where the roles that are implemented, we will identify studies in acute care and primary healthcare settings. Acute care will be defined as in-hospital or specialized ambulatory care to address specific health conditions [15]. Primary care will refer to the entry point of the healthcare system where patients receive comprehensive healthcare services for common health concerns [16].

Advanced practice nursing includes clinical and non-clinical activities related to education, research, and administration [17, 18]. According to the International Council of Nurses, APNs are nurses prepared at the graduate level who have acquired in-depth expertise, complex decision-making skills and advanced clinical competencies [2]. Master’s or doctoral educational preparation is recommended and in many countries is required with national board certification for licensure and entry-level practice [2]. Given the diversity of terms used globally to identify APNs, NPs, and CNSs, members of the research team will help identify role titles specific to their region. For example, CNSs may be identified as nurse consultants in some regions in the United Kingdom. We will be attentive to the countries and geographical distribution of the systematic reviews that are identified and adjust our search strategy as needed.

NPs are autonomous clinicians who practice in ambulatory, acute and long-term care as primary and/or specialty care providers, both independently and in coordination with healthcare professionals and others. NPs assess, diagnose, treat, and manage acute episodic and chronic illnesses. NPs are experts in health promotion and disease prevention. They order, conduct, supervise, and interpret diagnostic and laboratory tests, prescribe pharmacological agents and non-pharmacologic therapies, as well as teach and counsel patients, among other services. In addition to clinical practice, they may serve as healthcare researchers, interdisciplinary consultants, and patient advocates. NPs provide a wide range of services to individuals, families, groups, and communities [3]. For nurses to be considered as NPs in our review of reviews, the review must specify that they completed a formal post-baccalaureate or graduate NP education program.

CNSs have expertise in a nursing specialty and perform a role that includes practice, consultation, collaboration, education, research and leadership. CNSs assist in providing solutions for complex healthcare issues and are leaders in the development of clinical practice guidelines, promoting the use of evidence, and facilitating system change [2]. CNSs specialize in a specific area of practice that may be defined in terms of a population, setting, disease or medical subspecialty, type of care or type of problem. For nurses to be considered as CNSs, the review has to specify that they completed a graduate degree and the role described must be reflective of the CNS role definition.

#### Types of comparators

We will extract data related to the comparator (i.e., control) group to provide a brief description of the group to which care is being compared. Comparator groups can include the following, among others: usual care, best care, care provided by other healthcare professionals (e.g., physicians), or adherence to clinical practice guidelines.

#### Types of outcomes

The outcomes of interest for this review of reviews will include any outcome of an advanced practice nursing role. We will document measures at the levels of the patient (e.g., health status, patient satisfaction, quality of life), the provider (e.g., job satisfaction, quality of care), the health system (e.g., costs, length of hospital stay, rehospitalisation, resource utilisation), education, or policy/scope of practice. Outcomes will be categorized as clinical, provider, health system, educational, policy/scope of practice.

### Exclusion criteria

We will exclude reviews developed to address broad research questions (e.g., integrative reviews, literature reviews, scoping reviews).

We will exclude from the review of reviews studies related to physician assistants. Certified registered nurse anesthetists are excluded because, as of yet, they do not have global APN presence in the majority of countries with APN roles. We will also exclude nurse midwives since, across the different countries, not all regulatory requirements require these roles to be filled by nurses and nor are these roles consistently identified as advanced practice nursing roles. In reviews that include a mix of APN, NP and CNS roles and other provider roles, we will extract only data related to APNs, NPs and CNSs.

Moreover, we will exclude reviews where the impact of the APNs, NPs or CNSs cannot be teased out and is not reported separately from that of other types of nurses or healthcare team members. We will develop a list of all excluded reviews, along with the reasons justifying their exclusion.

### Database search

We will limit our search to January 2011 onwards to capture the most up-to-date trends, as evidence is outdated after five years in about half of published reviews [19]. We will search the following electronic databases: CINAHL, EMBASE, Global Health, HealthStar, PubMed, Medline, Cochrane Library Database of Systematic Reviews and Controlled Trials Register, Database of Abstracts of Reviews of Effects (DARE), Joanna Briggs Institute, and Web of Science. We will combine subject headings and keywords related to advanced practice nursing (e.g.: advanced practice nursing, nurse-led), APN (e.g., advanced practice nurse, advanced practice clinician, advanced practitioner, nurse prescriber), NP (e.g., nurse practitioner, advanced practice registered nurse, family nurse practitioner, primary healthcare nurse practitioner, adult gerontology nurse practitioner, pediatric nurse practitioner, oncology nurse practitioner, emergency nurse practitioner, mental health nurse practitioner, neonatal nurse practitioner), and CNS (e.g., nurse specialists, clinical nurse specialist, infection control practitioner, nurse consultant, specialist nurse) roles/titles, along with a search filter based on the CADTH systematic reviews and meta-analyses search filter and that developed by Lunny et al. for reviews of systematic reviews to capture a broad range of roles across settings [20, 21]. Subject headings and keywords will also include more general roles/titles, as well as those specific to primary and acute care settings, and corresponding acronyms where applicable. The full preliminary search strategy developed for the PubMed database, which will subsequently be adapted to each electronic database, is presented in S1 Appendix. We will adapt strategies reviewed by an academic librarian that have been used successfully in previous reviews [21]. In addition, we will hand search the reference lists of all relevant reviews to identify additional studies.

Moreover, we will search the grey literature will for the period of January 2011 onwards using the following websites and tools: World Health Organization, Organization for Economic Co-operation and Development (OECD), International Council of Nurses, CADTH Information Services, CADTH Grey Matters Tool, and ProQuest Dissertation and Theses. We will search the PROSPERO International Prospective Register of Systematic Reviews to identify registered review protocols, and will contact authors of registered PROSPERO reviews to ascertain study status. For each website, the content will be searched using the same search terms as those used for the published literature, e.g.: (Advanced practice nurs* OR Nurse practitioner* OR Clinical nurse specialist*) AND (Primary care OR Acute care) AND Systematic review*. If there is not an inherent search function on the website, a search will be conducted of all webpages and weblinks. The preliminary search strategy for the grey literature is presented in S2 Appendix.

### Study selection

To enhance inter-rater agreement, all reviewers will be trained to use the screening instrument and inclusion/exclusion criteria. We will upload the retained studies into the EndNote and RAYYAN software [22], after which duplicates will be removed. Two reviewers will independently screen titles and abstracts using the predefined inclusion/exclusion criteria, and recommend exclusion or further full-text review. Any discrepancies will be discussed among the reviewers. Inter-rater agreement will be estimated using the kappa statistic. Additional training sessions will be planned if inter-rater agreement is low and Cohen’s kappa is below 60% [23].

To be included in our review of reviews, each paper must be identified as a systematic review, and focus on an advanced practice nursing role or intervention. If the abstract contains insufficient information or there is no abstract available, we will complete a full-text review. We will complete a full-text review for all the reviews retained after the initial screening, again using the predefined inclusion/exclusion criteria. Any coding discrepancies will be discussed among the reviewers until agreement is reached on the inclusion or exclusion of the review. In the event they are unable to reach a consensus, a third reviewer will act as tie-breaker.

### Data extraction

Data from included full-text papers will be extracted by one coder and subsequently reviewed by a second coder. Any discrepancies will be resolved by consensus. A structured tool developed for a previous review of reviews will be adapted and pilot-tested by the investigators [12]. We will extract data from the methods and results section of each full-text paper. The data we will extract will include: review aim or focus; review characteristics (e.g., publication year); name and number of electronic databases searched; participant and intervention characteristics; number and types of studies included in the review; countries where studies were conducted; specification of patient, provider, health system, educational, policy, and scope of practice outcomes; and funding source [24]. Additionally, we will document APN, NP or CNS and non-APN involvement in the research team who conducted the review by extracting data related to the professional designation of the research team members.

### Design of included studies

Because the addition of APNs, NPs and CNSs is a complex healthcare system intervention, different types of information are needed to inform research about advanced practice nurses [25]. Systematic reviews included in our review of systematic reviews may include the results of randomized controlled trials, prospective controlled observational studies and cohort studies, retrospective controlled observational and cohort studies, and surveys. We will develop a summary table to present key findings.

### Assessment of review quality

Two reviewers will independently rate each systematic review using the 10-item Critical Appraisal Skills Programme (CASP) criteria [26] to assess the systematic review’s methodologic quality. As described above, inter-rater agreement will be assessed using Cohen’s kappa, and any disagreements will be discussed among the reviewers until they come to a consensus. We will generate a summary table with the CASP ratings.

### Outcomes

The primary outcome of the review of reviews is to document APNs, NPs or CNSs research globally to identify gaps in current research. We will examine each advanced practice nursing role separately.

### Data synthesis

A narrative synthesis of the findings will be compiled. We will use an iterative process to identify patterns and relationships emerging across the different reviews and years when they were conducted [27]. We will develop summary tables outlining the key review characteristics (e.g., publication year, countries where primary studies were conducted), outcomes (i.e., patient, provider, health system, educational, policy/scope of practice), type of advanced practice nursing role, and quality assessment. We will keep a record of all review-related decisions. No additional quantitative analyses are planned as this is not recommended for overviews because of the potential risk of overlap in studies that appear in more than one review [28].

## Discussion

The identification of advanced practice nursing roles that are currently in place, the countries where these nurses practice and the outcomes being used to examine practice will shed light on current gaps in the literature, and identify stronger and weaker areas of evidence related to advanced practice nursing globally. The review of systematic reviews builds on a recently completed umbrella review of NPs in primary healthcare. The current review of reviews will synthesize the characteristics of advanced practice nursing roles, study populations, contexts and outcomes to determine how closely these roles align with ICN definitions. In contexts where the roles are not optimally implemented or utilized, the findings will support the development of recommendations at the clinical, educational, and regulatory levels to improve role clarity, role implementation and access to high quality care. In addition, the development of an international strategic plan for APN role development will aid countries hoping to further expand APN practice.

## Supporting information

S1 Checklist PRISMA-P 2015 checklist. (PDF)

S1 Appendix Preliminary search strategies (PubMed) for the published literature. (PDF)

S2 Appendix Preliminary search strategies for the grey literature. (PDF)
