# Supplementary material for: A global perspective of advanced practice nursing research: A review of systematic reviews protocol
Source: PLoS One. 2023 Jan 24;18(1):e0280726. doi: 10.1371/journal.pone.0280726 (PMC9873152; doi:10.1371/journal.pone.0280726)
Supplement: S1 Appendix — (PDF) [file pone.0280726.s002.pdf]

S1 Appendix. Preliminary search strategies (PubMed) for the published literature

Title: A global perspective of advanced practice nursing research: A review of systematic reviews

Question: Do current systematic reviews that include advanced practice nurses (APNs), nurse practitioners (NPs) or clinical nurse specialists (CNSs) represent countries where these roles are found globally?

Aims: 1- Identify the countries included in systematic reviews of APNs, NPs or CNSs;
2- Describe the types of included studies, study population, role definitions, and context of care identified in the systematic reviews; and
3- Examine the types of outcomes of APN, NP or CNS roles included in systematic reviews globally

Search Strategy:

P: Participants will include patients and providers.
Patients of any age, health condition, groups or communities receiving care from an APN, NP or CNS in all types (e.g., public/private; teaching/non-teaching,), sizes (e.g., small/medium/large) and locations (e.g., urban/rural) of community or care agencies (e.g., acute, long-term care, primary care, home care) will be retained. Providers will include all members of the healthcare team in all types, sizes, and locations of organizations.

I: Interventions provided by APNs, NPs or CNSs in all sectors.
= Search strategy concept 1: Advanced Practice Nursing, comprised of the following 3 sub-concepts: 1) APN; 2) NP; 3) CNS.

C: Comparator (i.e., control) groups can include the following, among others: usual care, best care, care provided by other healthcare professionals (e.g., physicians), or adherence to clinical practice guidelines.

O: Any outcome of an advanced practice nursing role at the levels of the patient, the provider, the health system, education, or policy/scope of practice.

S: All relevant published and unpublished systematic reviews reported from January 2011 onwards, with no restrictions on jurisdiction or language.
= Search strategy concept 2: Search filter based on the CADTH systematic reviews and meta-analyses search filter and that developed by Lunny et al., 2016 for reviews of systematic reviews to capture a broad range of roles across settings.

(("Advanced Practice Nursing"[MH] OR
"advanced practice nurse"[tiab] OR
"advanced practice nurses"[tiab] OR
"advanced practice nursing"[tiab] OR
("APN"[tiab] AND "nurse"[tiab]) OR
("APN"[tiab] AND "nurses"[tiab]) OR
("APN"[tiab] AND "nursing"[tiab]) OR
"advanced nursing practice"[tiab] OR
"advanced nursing practices"[tiab] OR
"Nurse-led"[tiab] OR
"Nurses-led"[tiab] OR
"Nursing-led"[tiab] OR
"Advanced clinical practitioner"[tiab] OR
"Advanced clinical practitioners"[tiab] OR
"Advanced clinical practice"[tiab] OR
"Advanced practice clinician"[tiab] OR
"Advanced practice clinicians"[tiab] OR
"Advanced practitioner"[tiab] OR
"Advanced practitioners"[tiab] OR
Certified paediatric nurse[tiab] OR
Certified paediatric nurses[tiab] OR
"Certified pediatric nurse"[tiab] OR
"Certified pediatric nurses"[tiab] OR
"Community health nurse"[tiab] OR
"Community health nurses"[tiab] OR
"Community health nursing"[tiab] OR
"Expert nurse"[tiab] OR
"Expert nurses"[tiab] OR
"Expert nursing"[tiab] OR
("Master"[tiab] AND "advanced practice nursing"[tiab]) OR
("Nurse"[tiab] AND "advanced practice"[tiab]) OR
("Nurses"[tiab] AND "advanced practice"[tiab]) OR
"Nurse prescriber"[tiab] OR
"Nurse prescribers"[tiab] OR
Registered nurse extended class[tiab] OR
Registered nurses extended class[tiab] OR
"RN(EC)"[tiab] OR
Enfermera practica avanzada[tiab] OR
Enfermeras practica avanzada[tiab] OR
"Enfermera de practica avanzada"[tiab] OR
"Enfermeras de practica avanzada"[tiab] OR
"Enfermera gestora de casos"[tiab] OR
Enfermeras gestoras de casos[tiab] OR
"Infirmière de pratique avancée"[tiab] OR
Infirmières de pratique avancée[tiab] OR
Infirmiere de pratique avancee[tiab] OR
Infirmieres de pratique avancee[tiab] OR
"Infirmière en pratique avancée"[tiab] OR

Infirmières en pratique avancée[tiab] OR
Infirmiere en pratique avancee[tiab] OR
Infirmieres en pratique avancee[tiab] OR
Pflegeexperte APN[tiab] OR
"Pflegeexpertin"[tiab])
OR
("Nurse Practitioners"[MH] OR
"advanced nurse practitioner"[tiab] OR
"advanced nurse practitioners"[tiab] OR
("ANP"[tiab] AND "nurse"[tiab]) OR
("ANP"[tiab] AND "nurses"[tiab]) OR
("ANP"[tiab] AND "nursing"[tiab]) OR
"advanced practice registered nurse"[tiab] OR
"advanced practice registered nurses"[tiab] OR
"advanced practice registered nursing"[tiab] OR
("APRN"[tiab] AND "nurse"[tiab]) OR
("APRN"[tiab] AND "nurses"[tiab]) OR
("APRN"[tiab] AND "nursing"[tiab]) OR
"Nurse practitioner"[tiab] OR
"Nurse practitioners"[tiab] OR
("NP"[tiab] and "nurse"[tiab]) OR
("NP"[tiab] and "nurses"[tiab]) OR
("NP"[tiab] and "nursing"[tiab]) OR
"Advanced registered nurse practitioner"[tiab] OR
"Advanced registered nurse practitioners"[tiab] OR
"Certified nurse practitioner"[tiab] OR
"Certified nurse practitioners"[tiab] OR
"Certified registered nurse practitioner"[tiab] OR
"Certified registered nurse practitioners"[tiab] OR
"Medical nurse practitioner"[tiab] OR
Medical nurse practitioners[tiab] OR
Registered advanced nurse practitioner[tiab] OR
Registered advanced nurse practitioners[tiab] OR
Registered nurse extended class nurse practitioner[tiab] OR
Registered nurse extended class nurse practitioners[tiab] OR
"Registered nurse practitioner"[tiab] OR
"Registered nurse practitioners"[tiab] OR
Registered nurse-nurse practitioner[tiab] OR
Registered nurse-nurse practitioners[tiab] OR
"RN(NP)"[tiab] OR
"Women's health nurse practitioner"[tiab] OR
"Women's health nurse practitioners"[tiab] OR
("WHNP"[tiab] and "nurse"[tiab]) OR
("WHNP"[tiab] and "nurses"[tiab]) OR
("WHNP"[tiab] and "nursing"[tiab]) OR
"Infirmière praticienne spécialisée"[tiab] OR
Infirmières praticiennes spécialisées[tiab] OR
Infirmiere praticienne specialisee[tiab] OR

Infirmieres praticiennes specialisees[tiab] OR
"Family Nurse Practitioners"[MH] OR
"Primary healthcare nurse practitioner"[tiab] OR
"Primary healthcare nurse practitioners"[tiab] OR
"Primary health care nurse practitioner"[tiab] OR
"Primary health care nurse practitioners"[tiab] OR
"Primary health-care nurse practitioner"[tiab] OR
"Primary health-care nurse practitioners"[tiab] OR
("PHCNP"[tiab] AND "Nurse"[tiab]) OR
("PHCNP"[tiab] AND "Nurses"[tiab]) OR
("PHCNP"[tiab] AND "Nursing"[tiab]) OR
"Primary care nurse practitioner"[tiab] OR
"Primary care nurse practitioners"[tiab] OR
("PCNP"[tiab] AND "Nurse"[tiab]) OR
("PCNP"[tiab] AND "Nurses"[tiab]) OR
("PCNP"[tiab] AND "Nursing"[tiab]) OR
"Family nurse practitioner"[tiab] OR
"Family nurse practitioners"[tiab] OR
("FNP"[tiab] AND "Nurse"[tiab]) OR
("FNP"[tiab] AND "Nurses"[tiab]) OR
("FNP"[tiab] AND "Nursing"[tiab]) OR
"Geriatric nurse practitioner"[tiab] OR
"Geriatric nurse practitioners"[tiab] OR
"Gerontological nurse practitioner"[tiab] OR
"Gerontological nurse practitioners"[tiab] OR
("GNP"[tiab] AND "Nurse"[tiab]) OR
("GNP"[tiab] AND "Nurses"[tiab]) OR
("GNP"[tiab] AND "Nursing"[tiab]) OR
"Adult gerontology nurse practitioner"[tiab] OR
Adult gerontology nurse practitioners [tiab] OR
("AGNP"[tiab] AND "Nurse"[tiab]) OR
("AGNP"[tiab] AND "Nurses"[tiab]) OR
("AGNP"[tiab] AND "Nursing"[tiab]) OR
"Adult gerontology primary care nurse practitioner"[tiab] OR
"Adult gerontology primary care nurse practitioners"[tiab] OR
("AGPCNP "[tiab] AND "Nurse"[tiab]) OR
("AGPCNP "[tiab] AND "Nurses"[tiab]) OR
("AGPCNP "[tiab] AND "Nursing"[tiab]) OR
"Pediatric Nurse Practitioners"[MH] OR
"Adult gerontology acute care nurse practitioner"[tiab] OR
"Adult gerontology acute care nurse practitioners"[tiab] OR
("AGACNP"[tiab] AND "Nurse"[tiab]) OR
("AGACNP"[tiab] AND "Nurses"[tiab]) OR
("AGACNP"[tiab] AND "Nursing"[tiab]) OR
"Advanced critical care practitioner"[tiab] OR
"Advanced critical care practitioners"[tiab] OR
("ACCP"[tiab] AND "Nurse"[tiab]) OR
("ACCP"[tiab] AND "Nurses"[tiab]) OR

("ACCP"[tiab] AND "Nursing"[tiab]) OR
"Advanced neonatal nurse practitioner"[tiab] OR
"Advanced neonatal nurse practitioners"[tiab] OR
("ANNP"[tiab] AND "Nurse"[tiab]) OR
("ANNP"[tiab] AND "Nurses"[tiab]) OR
("ANNP"[tiab] AND "Nursing"[tiab]) OR
"Advanced paediatric nurse practitioner"[tiab] OR
Advanced paediatric nurse practitioners[tiab] OR
("cAPNP"[tiab] AND "Nurse"[tiab]) OR
("cAPNP"[tiab] AND "Nurses"[tiab]) OR
("cAPNP"[tiab] AND "Nursing"[tiab]) OR
Advanced pediatric nurse practitioner[tiab] OR
Advanced pediatric nurse practitioners[tiab] OR
Enhanced neonatal nurse practitioner[tiab] OR
Enhanced neonatal nurse practitioners[tiab] OR
("ENNP"[tiab] AND "Nurse"[tiab]) OR
("ENNP"[tiab] AND "Nurses"[tiab]) OR
("ENNP"[tiab] AND "Nursing"[tiab]) OR
"Oncology nurse practitioner"[tiab] OR
"Oncology nurse practitioners"[tiab] OR
("ONP"[tiab] AND "Nurse"[tiab]) OR
("ONP"[tiab] AND "Nurses"[tiab]) OR
("ONP"[tiab] AND "Nursing"[tiab]) OR
Pediatric acute care nurse practitioner[tiab] OR
Pediatric acute care nurse practitioners[tiab] OR
Paediatric acute care nurse practitioner[tiab] OR
Paediatric acute care nurse practitioners[tiab] OR
("PNPAC"[tiab] AND "Nurse"[tiab]) OR
("PNPAC"[tiab] AND "Nurses"[tiab]) OR
("PNPAC"[tiab] AND "Nursing"[tiab]) OR
"Acute care nurse practitioner"[tiab] OR
"Acute care nurse practitioners"[tiab] OR
("ACNP"[tiab] AND "Nurse"[tiab]) OR
("ACNP"[tiab] AND "Nurses"[tiab]) OR
("ACNP"[tiab] AND "Nursing"[tiab]) OR
"Adult nurse practitioner"[tiab] OR
"Adult nurse practitioners"[tiab] OR
"Critical care nurse practitioner"[tiab] OR
"Critical care nurse practitioners"[tiab] OR
"Emergency nurse practitioner"[tiab] OR
"Emergency nurse practitioners"[tiab] OR
("ENP"[tiab] AND "Nursing"[tiab]) OR
("ENP"[tiab] AND "Nurse"[tiab]) OR
("ENP"[tiab] AND "Nurses"[tiab]) OR
("Hospital-based"[tiab] AND "nurse practitioner"[tiab]) OR
("Hospital-based"[tiab] AND "nurse practitioners"[tiab]) OR
("Hospitalised"[tiab] AND "nurse practitioner"[tiab]) OR
("Hospitalised"[tiab] AND "nurse practitioners"[tiab]) OR

("Hospitalized"[tiab] AND "nurse practitioner"[tiab]) OR
("Hospitalized"[tiab] AND "nurse practitioners"[tiab]) OR
("Intensive care unit"[tiab] AND "nurse practitioner"[tiab]) OR
("Intensive care unit"[tiab] AND "nurse practitioners"[tiab]) OR
("Intensive care units"[tiab] AND "nurse practitioner"[tiab]) OR
("Intensive care units"[tiab] AND "nurse practitioners"[tiab]) OR
("ICU"[tiab] AND "nurse practitioner"[tiab]) OR
("ICU"[tiab] AND "nurse practitioners"[tiab]) OR
("ICUs"[tiab] AND "nurse practitioner"[tiab]) OR
("ICUs"[tiab] AND "nurse practitioners"[tiab]) OR
"Mental Health Nurse Practitioner"[tiab] OR
"Mental Health Nurse Practitioners"[tiab] OR
("MHNP"[tiab] AND "Nurse"[tiab]) OR
("MHNP"[tiab] AND "Nurses"[tiab]) OR
("MHNP"[tiab] AND "Nursing"[tiab]) OR
"Psychiatric-Mental Health Nurse Practitioner"[tiab] OR
"Psychiatric-Mental Health Nurse Practitioners"[tiab] OR
"Psychiatric Mental Health Nurse Practitioner"[tiab] OR
"Psychiatric Mental Health Nurse Practitioners"[tiab] OR
"PMHNP"[tiab] OR
"PMHNPs"[tiab] OR
"Pediatric nurse practitioner"[tiab] OR
"Pediatric nurse practitioners"[tiab] OR
"Paediatric nurse practitioner"[tiab] OR
"Paediatric nurse practitioners"[tiab] OR
("PNP"[tiab] AND "Nurse"[tiab]) OR
("PNP"[tiab] AND "Nurses"[tiab]) OR
("PNP"[tiab] AND "Nursing"[tiab]) OR
"Neonatal nurse practitioner"[tiab] OR
"Neonatal nurse practitioners"[tiab] OR
("NNP"[tiab] AND "Nurse"[tiab]) OR
("NNP"[tiab] AND "Nurses"[tiab]) OR
("NNP"[tiab] AND "Nursing"[tiab]))
OR
("Nurse Specialists"[MH] OR
"Infection Control Practitioners"[MH] OR
"Clinical nurse specialist"[tiab] OR
"Clinical nurse specialists"[tiab] OR
("CNS"[tiab] AND "nurse"[tiab]) OR
("CNS"[tiab] AND "nurses"[tiab]) OR
("CNS"[tiab] AND "nursing"[tiab]) OR
"Clinical nurse consultant"[tiab] OR
"Clinical nurse consultants"[tiab] OR
"Clinical specialist"[tiab] OR
"Clinical specialists"[tiab] OR
"Infection control practitioner"[tiab] OR
"Infection control practitioners"[tiab] OR
"Nurse clinician"[tiab] OR

"Nurse clinicians"[tiab] OR
"Nurse consultant"[tiab] OR
"Nurse consultants"[tiab] OR
"Nurse specialist"[tiab] OR
"Nurse specialists"[tiab] OR
"Specialist nurse"[tiab] OR
"Specialist nurses"[tiab] OR
Infirmière clinicienne spécialisée[tiab] OR
Infirmières cliniciennes spécialisées[tiab] OR
Infirmiere clinicienne specialisee[tiab] OR
Infirmieres cliniciennes specialisees[tiab] OR
"Verpleegkundig specialist"[tiab] OR
Verpleegkundig specialist geestelijk gezondheidszorg[tiab] OR
Verpleegkundig specialist acute zorg bij somatische aandoeningen[tiab]))
AND
(("systematic"[filter] OR
"meta-analysis"[pt] OR
"meta-analysis as topic"[mh] OR
meta analy*[tw] OR
metanaly*[tw] OR
metaanaly*[tw] OR
met analy*[tw] OR
research overview*[tiab] OR
"systematic review"[pt] OR
"systematic reviews as topic"[mh] OR
systematic review*[tiab]) OR
((overview*[ti] OR review[ti] OR synthesis[ti] OR summary[ti] OR cochrane[ti] OR analysis[ti]) AND
(reviews[ti] OR meta-analyses[ti] OR articles[ti])) OR
(meta-review[tiab] OR meta review[tiab]) OR
((overview*[ti] OR reviews[ti]) AND (systematic[ti] OR cochrane[ti])) OR
(reviews[tiab] AND (meta [tiab] OR published[tiab] OR quality[tiab] OR included[tiab] OR
summar*[tiab])) OR
("cochrane reviews"[tiab]) OR
(evidence[ti] AND (reviews[ti] OR meta-analyses[ti])))
